# Idealistic, realistic, and unrealistic expectations of pharmacological treatment in persons with type 2 diabetes in primary care

**DOI:** 10.3389/fpubh.2023.1058828

**Published:** 2023-02-02

**Authors:** Ana María Salinas Martínez, Angélica Gabriela Juárez Montes, Yesenia Ramírez Morado, Hid Felizardo Cordero Franco, Francisco Javier Guzmán de la Garza, Luis Carlos Hernández Oyervides, Georgina Mayela Núñez Rocha

**Affiliations:** ^1^Universidad Autónoma de Nuevo León, Facultad de Salud Pública y Nutrición, Monterrey, Mexico; ^2^Epidemiologic and Health Services Research Unit/CIBIN, Mexican Institute of Social Security, Monterrey, Mexico; ^3^Family Medicine Clinic and General Hospital of Subzone No. 12, Mexican Institute of Social Security, Linares, Mexico; ^4^Universidad Autónoma de Nuevo León, Facultad de Medicina, Monterrey, Mexico

**Keywords:** expectations, diabetes, primary care, therapeutic misconception, individual preference

## Abstract

**Introduction:**

Information on treatment expectations in diabetes is scarce for Mexican and Latino populations. We determined idealistic, realistic, and unrealistic expectations for metformin, insulin, and glyburide in primary care. We also explored the association between sociodemographic attributes, time since diagnosis, and expectations.

**Methods:**

This was a cross-sectional study conducted during 2020–2022 in governmental primary care centers. We consecutively included persons with type 2 diabetes aged 30–70 years under pharmacological medication (*n* = 907). Questions were developed using information relevant to expectation constructs. Data were collected by interview. We used descriptive statistics, a test of the difference between two proportions, and multivariate ordinal logistic regression.

**Results:**

A high percentage of participants would like to have fewer daily pills/injections or the option of temporarily stopping their medication. Realistic expectations ranged from 47% to 70%, and unrealistic expectations from 31 to 65%. More insulin users wished they could take a temporary break (*p* < 0.05) or would like to be able to change the route of administration (*p* < 0.001) than metformin users. More persons with diabetes on insulin expected realistic expectations compared to those on metformin or glyburide (*p* ≤ 0.01). Being able to interrupt medication upon reaching the glucose goal was higher in combined therapy users (*p* < 0.001).

**Conclusion:**

Time since diagnosis, place of residence, sex, and diabetes education were factors associated to expectations. Management of expectations must be reinforced in primary care persons with type 2 diabetes undergoing pharmacological medication.

## 1. Introduction

Diabetes is a chronic condition characterized by high blood glucose levels, which, if left untreated, leads to heart, vascular, eye, kidney, and nerve complications. There are two main types of diabetes: type 2 and type 1. The first is initially due to insulin resistance that progresses to loss of adequate insulin secretion by b-cells. Type 1 is an insulin-dependent diabetes, in which the pancreas produces little or no insulin by itself due to autoimmune b-cell destruction (1, 2). Type 2 diabetes is the most common type accounting for over 90% of all diabetes cases worldwide. An estimated 537 million adults aged 20–79 years were living with diabetes in 2021 (10.5% prevalence); 50.5 million in the North America and Caribbean region (14.0% prevalence) and 32.5 million in the South and Central America region (9.5% prevalence). Mexico is among the top 10 countries for diabetes, with 14.1 million people affected (15% prevalence) (2). Regular physical activity, a healthy diet, and a healthy body weight are key factors that must be promoted by public and private health institutions since they are the foundation of type 2 diabetes management. If the adoption of healthy habits does not achieve optimal glucose levels, pharmacotherapy should be initiated. Metformin is one of the first-choice oral medications. If a single diabetes-specific medication does not work, a range of combination therapy options are available (sulfonylureas, thiazolidinediones, and alpha glucosidase inhibitors, among others). Additionally, insulin injections may be needed to reach glycemic goals (3, 4). From 25 to 90% of persons with type 2 diabetes do not take medication as prescribed (5). Differences in managing diabetes expectations between the diabetes care team and persons living with diabetes lead to inconsistencies in taking medication because those who notice no benfit may stop taking them (6). Meeting expectations is important because the individual may think medication does not work (7), and dissatisfied persons are less likely to follow the medication plan or to take an active role in their care (8). The loss of continuity in taking the medication will, in turn, decrease therapeutic efficacy, with consequences on target glucose levels (9–11), hospitalization (5, 10), mortality (5, 12), and high costs (10, 13). Therefore, awareness of personal expectations is essential for healthcare professionals, health managers, and health policymakers.

An expectation refers to the anticipation of the occurrence of a specific outcome. It is a type of belief or perception of a future event. The theory of expectations in psychology maintains that they are the product of a cognitive process dependent on experience and social learning (14, 15). The individual compares what is anticipated with what is received and confirms or modifies his/her expectations. He/she also compares results with other individuals, medications, and health conditions (7, 16). There are structural (e.g., tablet shape and color) and process (e.g., medication procedure) expectations. For instance, an injectable medication may be anticipated to be more effective than an oral one, or a medicine prescribed by a cardiologist may be anticipated to be more effective than one prescribed by a general practitioner (15). Expectations can be idealistic (what the person wants or prefers if given the choice), unrealistic (myth or fantasy), or realistic (predictive). Moreover, realistic expectations can be positive (e.g., symptom relief, hospitalization prevention, goal achievement) or negative (e.g., adverse effect experience) (7, 14, 15, 17). The literature on realistic expectations show 70% of persons living with diabetes on oral medication anticipate the benefit of achieving target glucose levels and only 11.7% expect a reduction in the risk of complications (18). One study found that only one-fifth of persons with diabetes expected to take glucose-lowering agents for the rest of their lives (19). Moennig et al. (20) found that 42% of insulin users expected an improvement in glucose, and fulfillment of this expectation was the main cause of uninterrupted use. Naegeli et al. (21) documented a higher percentage of insulin users anticipate achieving optimal glucose levels (61%). Notably, 58% had their expectations exceeded and 29% their expectations fulfilled. On the other hand, the following erroneous expectations have been reported: being able to stop the medication when reaching the glucose goal, anticipating diabetes cure, and expecting freedom to eat while taking the medicine (18, 19, 22–26). The study of expectations in diabetes is worthwhile because persons living with diabetes with positive outcome expectations are more likely to benefit from diabetes management than those with negative outcome expectations (27), while idealistic expectations or misperceptions seem to have the opposite effect on health outcomes (15).

Personal expectations may vary according to ethnic origin, age, sex, socioeconomic status, schooling, diabetes education, time since diagnosis, and diabetes severity (14, 22, 28). However, information on expectations and associated factors in persons with diabetes is practically non-existent in Mexican or Latino populations (24). The objective of this study was to determine the idealistic, realistic, and unrealistic expectations of pharmacological medication (metformin, insulin, and glyburide) among persons with type 2 diabetes in primary care. We also explored the association between sociodemographic attributes (sex, schooling, place of residence, education in diabetes), time since diagnosis, and expectations.

## 2. Materials and methods

This was a cross-sectional study conducted from October 2020 to March 2022. We consecutively included persons with type 2 diabetes between 30 and 70 years old under pharmacological therapy. Those with current pregnancy or history of blindness, hemodialysis, peritoneal dialysis, lower-extremity amputation, and heart surgery were excluded (to limit persons with advanced diabetes severity among whom expectations might differ from those of persons with mild or no complications). The participants were approached in waiting rooms of primary care centers of a governmental health institution in the metropolitan area of Monterrey (the third-largest urban area of the country, with a population density of 3,523 inhabitants/km^2^) and one primary care center in a suburban area located in Linares (population density of 33.7 inhabitants/km^2^), Mexico. The prevalence of idealistic, realistic, and unrealistic expectations was taken as the parameter (*p*) for estimating the sample size. A minimum sample size of 385 individuals with diabetes was required considering *p* = 50% with a 95% confidence level and a precision of 5%. However, the total sample size was 907; 507 participants from the urban area and 400 participants from the suburban area. The protocol was approved by the Local Committees of Ethics and Health Research (No. 2020-1909-062, 2021-1909-101, and 20-FASPYN-SA-22.TP). Informed consent was provided by all the participants. Anonymity was always preserved, and the confidentiality of the data was ensured.

### 2.1. Study variables

Three types of expectations were studied: idealistic, realistic, and unrealistic; and the anticipation of a future event was the common definition. Idealistic expectations focused on management preferences, realistic expectations on perceived medication efficacy, and unrealistic expectations on incorrectly perceived therapeutic benefits. Expectation items were subject to content validity evaluation. A group of experts (three medical doctors and two public health specialists) assessed and approved by consensus the pertinence and relevance of the questions. Special attention was paid to avoiding ambiguity and technical vocabulary. Pre-test and pilot tests were carried out to verify clarity and ease of understanding. The internal consistency results are provided below.

#### 2.1.1. Idealistic expectations domain

Measurement was based on what the person with diabetes preferred if given the choice. Questions were developed using information relevant to idealistic expectation constructs (14, 15). Three items were included: (1) If you had the option, you would like to take fewer pills or receive fewer applications of insulin per day (in those who took more than one tablet or received more than one application per day); (2) You would like to be able to change the route of administration from oral to injectable, or vice versa; and (3) You wish you could take a temporary break from the medication. In persons treated with metformin, a fourth item on preference for a smaller tablet size was also included. The response options were on a Likert scale (−1 = No, 0 = Indifferent, 1 = Yes). The questions were specific to the medication being received, so an individual on metformin answered the questions about metformin. If someone was on two medications, for example, metformin and insulin, he/she answered the metformin and insulin questions separately (metformin: *n* = 725, Cronbach's Alpha = 0.97; insulin: *n* = 352, Cronbach's Alpha = 0.94; glyburide: *n* = 180, Cronbach's Alpha = 0.68). For the analysis of association, an index was constructed to summarize the idealistic expectations domain. The negative and indifferent responses were regrouped and coded as 0; the positive response remained as 1. Responses were then summed and categorized into null, low, and moderate-high depending on the number of idealistic expectations 0, 1, and 2–3, respectively.

#### 2.1.2. Realistic expectations domain

Measurement was based on the perception of true medication benefits. Six items adapted from other authors (7, 18) were used: (1) How much do you expect the medicine will bring blood sugar down to a normal range; (2) Eliminate symptoms of hyperglycemia; (3) Prevent or delay foot amputations; (4) Prevent or delay the need for dialysis; (5) Prevent or delay vision loss; and (6) Reduce the need for hospitalization. The response options were on a Likert scale (1= Null, 4= Very much). Cronbach's Alpha was as follows: Metformi*n* = 0.92, insuli*n* = 0.90, and glyburide: = 0.90. For the analysis of association, an index was constructed to summarize the realistic expectations domain. The “null”, “a little”, and “moderately” responses were regrouped and coded as 0; the “very much” response was recoded as 1. Responses were then summed and categorized into null, low, moderate, and high depending on the number of realistic expectations 0, 1–2, 3–4, and 5–6, respectively.

#### 2.1.3. Unrealistic expectations domain

Measurement was based on misperception of medication benefits. Four items adapted from other authors (22–25) were used: (1) How much do you expect the medicine will cure your diabetes; (2) Allow you to stop treatment when reaching your glucose goal; (3) Allow freedom to eat; and (4) Allow you to have no complications despite medication. The response options were on a Likert scale (1=Null, 4= Very much). The questions were generic; for example, a person treated with metformin and insulin answered the section without distinguishing between medications (*n* = 907, Cronbach's Alpha = 0.66). For the analysis of association, an index was constructed to summarize the unrealistic expectations domain. The “a little”, “moderately”, and “very much” responses were regrouped and coded as 1; the “null” response was recoded as 0. Responses were then summed and categorized into null, low, and moderate-high depending on the number of unrealistic expectations 0, 1, and 2–4, respectively.

#### 2.1.4. Other variables

Sociodemographic attributes (sex, age, marital status, schooling, occupation, place of residence), diabetes education in the last year (yes, no), time since diagnosis (years), and frequency of medical visits for diabetes management (monthly, every 2 months, other) were included. Comorbidities (hypertension, dyslipidemia, other) (yes, no), hospitalization due to diabetes in the past year (yes, no); and last glucose result (mg/dL) (self-report) were also considered. Data were collected through a face-to-face interview lasting approximately 15 min. Two postgraduate students (Family Medicine and Master of Sciences in Public Health) and two medical interns participated. All were supervised by the principal investigator. Participants with incorrect expectations were given the correct information at the end of the survey. Those with idealistic expectations were instructed to express their preferences to their doctor at the next visit, so that they could jointly analyze the possibility of satisfying them.

#### 2.1.5. Statistical analysis

Means and standard deviations were used to describe continuous variables, and percentages to describe categorical variables. The z test for the difference between two proportions was used for comparing the frequency of expectations by type of medication. The chi-square test (univariate analysis) and ordinal logistic regression (multivariate analysis) were employed for analyzing the association between factors under study and expectations; the sociodemographic attributes and time since diagnosis were considered the independent variables; the idealistic, realistic, or unrealistic expectations index constituted the dependent variable; and the comorbidity and frequency of medical visits for diabetes management were the control variables. Odds ratios (OR) and 95% confidence intervals (CI) were estimated for quantifying the strength of the association between associated factors and expectations.

## 3. Results

The mean age was 55.7 ± 10.6, and the mean time since diabetes diagnosis was 9.9 ± 7.9 years. Most of the participants were female and married or with a partner. More than 80% had routine monthly medical visits for diabetes management. Table 1 shows in detail the sociodemographic and comorbidity profile of the study population.

**Table 1:** Sociodemographic and comorbidity profile (*n* = 907).

**Attribute**	**Frequency**
Sex, female	62.3%
Marital status, married or with partner	71.3%
**Schooling**	
Primary	38.3%
Secondary	29.9%
High school and higher	31.9%
**Occupation**	
Employed	36.3%
Self-employed	6.3%
Housewife	39.3%
Retired	16.4%
Unemployed	1.7%
History of diabetes education, during last year	20.3%
Diabetes management medical visits, monthly	80.8%
Glucose <110 mg/dL (self-report)	18.8%
Hypertension	51.0%
Dyslipidemia	42.3%
Other (urinary, circulatory, vision, or neuropathy)	13.0%
History of hospitalization, during last year	1.8%

### 3.1. Idealistic expectations

**Figure 1:**
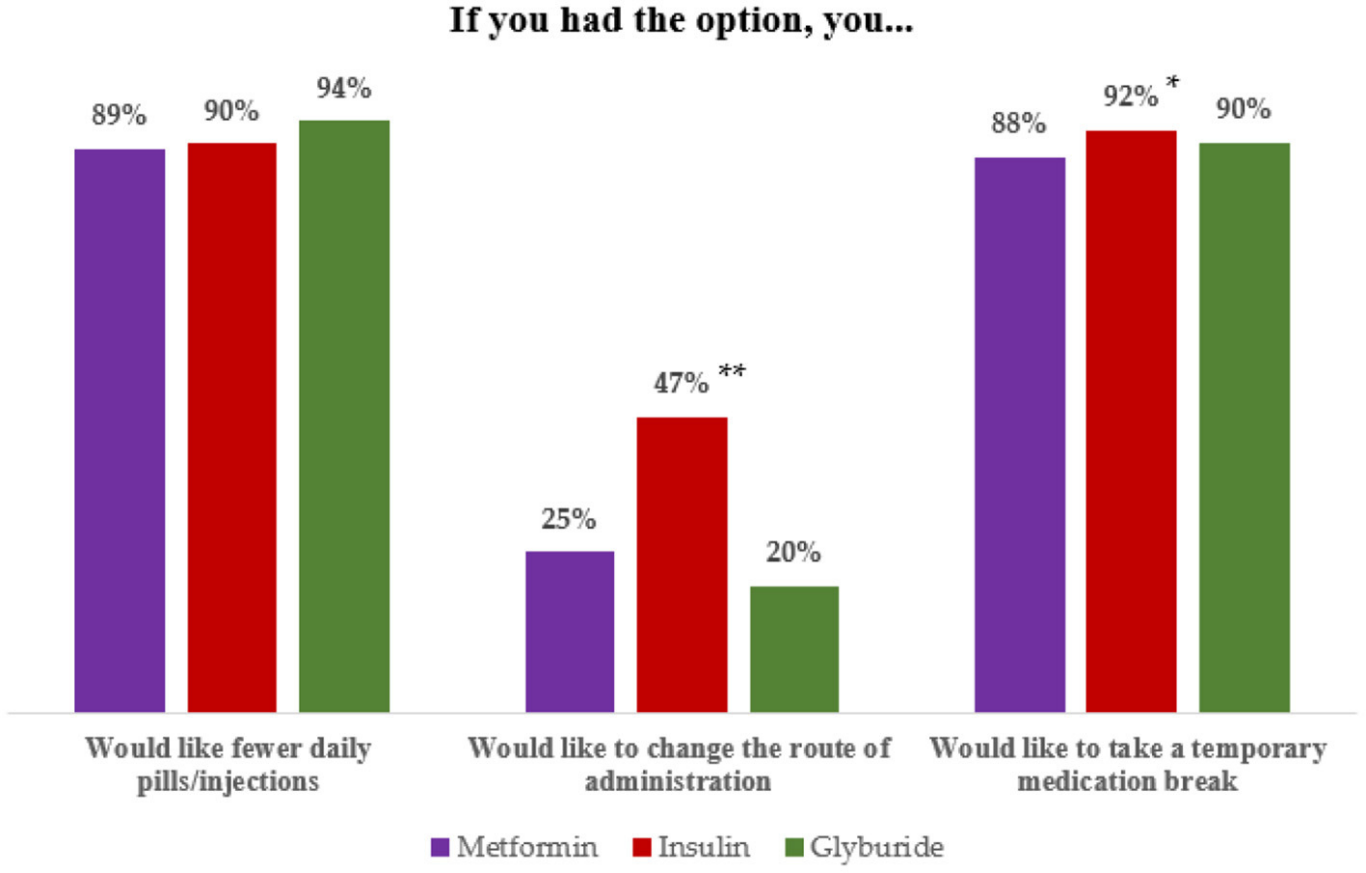
Idealistic expectations according to type of medication. Information at the bottom of the figure: z test for the difference between two proportions *p* values: * <0.05, Insulin > Metformin, ** <0.001, Insulin > Metformin and glyburide.

A high percentage of participants on metformin would like the pill size to be smaller (77.8%). About 90% would like fewer pills/injections per day. More insulin users wished they could take a temporary break or would like to change the route of administration (Figure 1). Suburban residence, ≥ 3 years since diagnosis, and female sex increased the odds of higher metformin idealistic expectations. Place of residence was also associated to glyburide idealistic expectations and time since diagnosis with those of insulin (Table 2).

**Table 2:** Multivariate ordinal regression analyses of factors associated to idealistic and realistic expectations.

	**Type of medication**		
	**Metformin**	**Insulin**	**Glyburide**
	**(*****n** =* **725)**	**(*****n** =* **352)**	**(*****n** =* **180)**
	**Adjusted odds ratios**^a^ **(95%CI)**		
**Idealistic expectations (moderate-high)** ^b^			
Residence, suburban	1.6 (1.1, 2.3)^**^	1.4 (0.7, 2.8)	3.5 (1.7, 7.3)^***^
Time since diagnosis, ≥ 3 years	2.7 (1.8, 3.9)^***^	5.5 (2.3, 13.1)^***^	0.6 (0.2, 1.7)
Sex, female	1.5 (1.1, 2.0)^*^	0.9 (0.5, 1.7)	1.1 (0.6, 2.2)
Comorbidity, hypertension and/or dyslipidemia	0.8 (0.5, 1.1)	1.3 (0.7, 2.3)	0.9 (0.4, 2.0)
**Realistic expectations (high)** ^c^			
Residence, suburban	1.9 (1.4, 2.6)^***^	1.6 (0.9, 2.6)	1.4 (0.8, 2.7)
Time since diagnosis, ≥3 years	0.9 (0.6, 1.3)	0.9 (0.4, 1.9)	0.5 (0.2, 1.3)
Sex, female	1.1 (0.8, 1.5)	1.3 (0.8, 1.9)	1.0 (0.6, 1.8)
Comorbidity, hypertension and/or dyslipidemia	1.4 (1.0, 1.8)^*^	1.3 (0.8, 2.0)	1.2 (0.6, 2.2)

Wald Chi-square *p* values: ^*^ < 0.05, ^**^ < 0.01, ^***^ < 0.001.

^a^Variables present in the model without statistical significance were schooling, diabetes education in the last year, and frequency of medical visits for diabetes management (*p* > 0.05). ^b^Index of 2-3 idealistic expectations with an affirmative response (out of a total of 3). ^c^Index of 5–6 realistic expectations with a “very much” response (out of a total of 6).

### 3.2. Realistic expectations

Between 47 and 70% of participants had realistic expectations; insulin users had more realistic expectations than metformin users (Table 3). Suburban residence and having hypertension and/or dyslipidemia increased the odds of higher metformin realistic expectations (Table 2).

**Table 3:** Realistic expectations according to type of medication.

	**Type of medication**		

**The medicine will very much…**	**Metformin**	**Insulin**	**Glyburide**
	**(*****n** =* **725)**	**(*****n** =* **352)**	**(*****n** =* **180)**
	**(A)**	**(B)**	**(C)**
Bring blood sugar down to a normal range	54%^**a^	70%^***b^	67%
Eliminate hyperglycemia symptoms	47%	59%^**b^	54%
Prevent/delay foot amputation	51%	61%^**b^	54%
Prevent/delay need for dialysis	49%	62%^**b^	50%^*c^
Prevent/delay vision loss	48%	59%^*b^	49%
Reduce need for hospitalization	52%	63%^**b^	57%

z test for the difference between two proportions *p* values: ^*^ ≤ 0.01, ^**^ < 0.001, ^***^ < 0.0001; ^a^A < C, ^b^B > A, ^c^C < B.

### 3.3. Unrealistic expectations

The most frequent unrealistic expectation was anticipating complications despite the medication (64.8%). It was followed by expecting interruption of medication upon reaching the glucose goal (55.0%), freedom to eat (39.1%), and diabetes cure (30.7%). Combined therapy users had a higher expectation of being able to discontinue the medication upon reaching the glucose goal (Table 4). Suburban residence increased 1.98 times (95%CI 1.47, 2.68) the odds of higher unrealistic expectations while diabetes education in the last year decreased them (OR 0.62, 95% CI 0.44, 0.87); after adjustment for type of medication, sex, schooling, time since diagnosis, comorbidity, and frequency of medical visits for diabetes management.

**Table 4:** Unrealistic expectations according to therapeutic plan.

	**Therapeutic plan**				
**The medicine will very much, moderately, a little…**	**Metformin**	**Insulin**	**Glyburide**	**Metformin** + **insulin**	**Metformin** + **Glyburide**
	**(*****n** =* **375)**	**(*****n** =* **162)**	**(*****n** =* **20)**	**(*****n** =* **190)**	**(*****n** =* **160)**
	**(A)**	**(B)**	**(C)**	**(D)**	**(E)**
Cure diabetes	33.1%	28.4%	20.0%	31.6%	27.7%
Allow stopping treatment when reaching glucose goal	50.9%	47.5%	25.0%	63.2%^**a^	66.3%^**a^
Allow freedom to eat	37.6%	35.8%	45.0%	41.1%	43.1%
Allow complications despite treatment	61.3%	66.7%	50.0%^§*c*^	64.7%	73.1%^*b^

z test for the difference between two proportions *p* values: ^*^ ≤ 0.01, ^**^ < 0.001, ^§^ = 0.06; ^a^D and E > A, B, and C, ^b^E > A, ^c^C < E.

## 4. Discussion

We determined idealistic, realistic, and unrealistic expectations about metformin, insulin, and glyburide in persons with type 2 diabetes in primary care. We found several expectation differences by type of medication. Almost half of the people on insulin would like to be able to switch to oral administration compared to one-fourth on metformin who would like the opposite, indicating that personal preferences should not be taken for granted. Boye et al. (29) evidenced 25.5% of persons with diabetes had switched from preferring oral to injectable medication after learning about the product specifications (daily oral semaglutide vs. once-weekly injectable dulaglutide). We also identified 8 out of 10 participants on metformin would like the size of the pill to be smaller and a high percentage would like fewer daily pills/injections or a temporary break from the medication. Fairchild et al. (19) documented 78% of adults in primary care did not expect to take oral medications for life. Idealistic expectations must be discussed with the person living with diabetes because the ideal may not be feasible. And personal preferences, shared decision making, and mutual agreement for changes should be encouraged.

There were more real benefits perceived with insulin than metformin. Laferton et al. (15) also showed an injectable medication was expected to be more effective than an oral one. Reduction of glucose to a target level was the number one most expected benefit, 7 out of 10 insulin participants anticipated this result, higher than 42 and 61% reported by multinational studies (20, 21). In contrast, 5 out of 10 metformin users expected this benefit, a result lower than that documented in primary care of 70% (18). Differences in findings emphasize the importance of identifying expectations in different populations. Correspondence between the diabetes care team members and the person with diabetes is essential since it may contribute to non-taking the medication by failing to explain benefits and side effects adequately (30). Realistic expectations must be reinforced, since the greater the perception of effectiveness, the greater the medication taking (31, 32). Furthermore, a low efficacy perception has been associated with higher levels of HbA1c (33).

More than half of the respondents anticipated complications would occur despite treatment or that they could stop the medication when the glucose goal was reached. In Saudi Arabia, these were also the most common unrealistic expectations (89 and 66.5%, respectively) (22). Analysis by type of therapeutic plan showed combined therapy users expected more to discontinue the medication upon reaching the glucose goal. One-third anticipated diabetes would be cured over time with no differences by medication scheme. The frequency of this erroneous expectation has been wide-ranging (11.7–65%) (19, 23–26) and some authors such as Mann et al. (25) have reported insulin users are less likely to believe in diabetes cure. Additionally, over a third of participants expected they could eat anything while taking the medication. Literature reports vary from 23 to 49.1% (23, 24, 26). Food freedom means always eating what you want in the amount you want. Freedom might be an exception, but not the rule. Medical nutrition therapy plays an integral role in diabetes management that considers maintaining the pleasure of eating by providing flexibility with healthy food choices, while limiting unnecessary and unhealthy ones. The American Diabetes Association advises people with diabetes to minimize the consumption of foods with added sugar and refined grains. It also emphasizes the consumption of polyunsaturated fats and limits the serving size of nutrient-dense foods for favoring a healthy body weight and achieving glucose and lipid goals (34). Certainly, knowledge and beliefs about medications should be attended to. Health educators and decision makers should keep in mind that outcome expectations and self-care behaviors are correlated (35, 36), and that unrealistic expectations constitute barriers to effective diabetes management. Clearly, the insertion of effective health communication strategies is urgently needed to neutralize misconceptions, such as the diabetes cure, and to reinforce true facts.

We explored the association between several factors and expectations, ≥3 years since diagnosis increased the odds of higher metformin and insulin idealistic expectations indicating need for matching correct information over time. Time since diagnosis was not associated to unrealistic expectations, which differed from other studies that have shown greater misperceptions with <5 (26) or between 5 and 15 years with diabetes (22). This lack of consistency requires further research. Being female increased the odds of metformin idealistic expectations, but not those of erroneous expectations. Other authors have identified women tend to have higher misconceptions (22, 26). Suburban residence was another associated factor. It augmented the odds of metformin idealistic and realistic expectations, also the odds of glyburide idealistic expectations, and those of unrealistic expectations. There are three ways to create expectations: direct personal experience, observation, and suggestion of others (27). Dissimilarity in such circumstances could explain differences between urban and non-urban residents, but more investigation is needed to identify the specific reasons. Diabetes education in the last year lessened the odds of misperceptions. Alsunni et al. (22) found individuals who had undergone proper education about diabetes had less misconceptions, underlining the importance of educational programs. Diabetes self-management education is essential in the care of all people with diabetes to provide knowledge and skills. And the 2022 National Standards for Diabetes Self-Management Education and Support recommend the collaboration between the person and the health care team considering the individual's concerns, needs and priorities (37).

### 4.1. Limitations

Obtaining socially acceptable responses could have led to overestimation or underestimation of expectations. This study focused on the most frequently employed diabetes medication, so dipeptidyl peptidase 4 inhibitors (DPP-4), thiazolidinediones, and sodium glucose co-transporter 2 inhibitors users were not considered. Only persons with diabetes from primary care without current pregnancy or advanced complications were included, so it is not possible to generalize results to those with gestational diabetes or under diabetes management in secondary or tertiary care. More research is needed, and future investigations should include these types of cases. More than half of the study population were women, which was not surprising. In Mexico, there are more women than men according to the 2020 population census and diabetes is more prevalent in women (38, 39). The association analysis had the advantage of being multivariate, but the study design was cross-sectional. Future longitudinal studies are required for definitive conclusions on factors determining idealistic, realistic, and unrealistic expectations.

## 5. Conclusions

This study contributes to narrowing the knowledge gap about idealistic, realistic, and unrealistic expectations in the Mexican or Latino population regarding pharmacological medication in persons with type 2 diabetes. The frequency varied by expectation and type of medication. Insulin users had more idealistic and realistic expectations; and combined therapy users expected more to discontinue the medication upon reaching the glucose goal. Time since diagnosis, place of residence, and sex were factors associated to expectations. Especially, diabetes education reduced the odds of misperceptions. Health policy makers, health managers, and the diabetes care team together must ensure that people with diabetes be trained. Understanding personal preferences and expectations is relevant because it makes it easier to select the medication that will most benefit the individual. A person-centered communication, shared decision making, and management of expectations must be reinforced in persons with type 2 diabetes undergoing pharmacological medication in primary care.

## Data availability statement

The original contributions presented in the study are included in the article/supplementary material, further inquiries can be directed to the corresponding author.

## Ethics statement

The studies involving human participants were reviewed and approved by Institutional Review Board and Ethics Committee of the Mexican Social Security Institute (No. 2020-1909-062 and 2021-1909-101) and the School of Public Health and Nutrition (20-FASPYN-SA-22.TP). The patients/participants provided their written informed consent to participate in this study.

## Author contributions

Conceptualization and methodology: AS and AJ. Data curation and investigation: AJ, YR, and LH. Formal analysis and validation: AS and HC. Project administration: AS, FG, and GN. Supervision and writing—original draft: AS. Writing—review and editing: AS, AJ, YR, HC, FG, LH, and GN. All authors contributed to the article and approved the submitted version.
